# Roles of host and environment in shift of primary anthrax host species in Kruger National Park

**DOI:** 10.1371/journal.pone.0314103

**Published:** 2024-12-06

**Authors:** Sunday O. Ochai, Lourens Snyman, Amelie C. Dolfi, Abel Ramoelo, Brian K. Reilly, Judith M. Botha, Edgar H. Dekker, O. Louis van Schalkwyk, Pauline L. Kamath, Emma Archer, Wendy C. Turner, Henriette van Heerden

**Affiliations:** 1 Department of Veterinary Tropical Diseases, Faculty of Veterinary Science, University of Pretoria, Onderstepoort, South Africa; 2 Antimicrobial Research Unit, College of Health Sciences, University of KwaZulu-Natal, Durban, South Africa; 3 Department of Geography, Geoinformatics and Meteorology, University of Pretoria, Hatfield, Pretoria, South Africa; 4 Department of Forest and Wildlife Ecology, Wisconsin Cooperative Wildlife Research Unit, University of Wisconsin-Madison, Madison, WI, United States of America; 5 Department of Genetics, Faculty of Natural and Agricultural Sciences, University of Free State, Bloemfontein, South Africa; 6 Savanna Research Unit, Scientific Services, South African National Parks, Skukuza, South Africa; 7 Department of Agriculture, Office of the State Veterinarian, Forestry and Fisheries, Government of South Africa, Skukuza, South Africa; 8 Department of Migration, Max Planck Institute of Animal Behavior, Radolfzell, Germany; 9 School of Food and Agriculture, University of Maine, Orono, Maine, United States of America; 10 Department of Forest and Wildlife Ecology, U.S. Geological Survey, Wisconsin Cooperative Wildlife Research Unit, University of Wisconsin-Madison, Madison, WI, United States of America; Universitat Autonoma de Barcelona, SPAIN

## Abstract

Environmental and climatic factors, as well as host demographics and behaviour, significantly influence the exposure of herbivorous mammalian hosts to pathogens such as *Bacillus anthracis*, the causative agent of anthrax. Until the early 1990s in Kruger National Park (KNP), kudu (*Tragelaphus strepsiceros*) was the host species most affected by anthrax, with outbreaks occurring predominantly in the dry season, particularly during drought cycles. However, the most affected host species has shifted to impala (*Aepyceros melampus*), with more frequent anthrax outbreaks during the wet season. This study investigates the roles of environmental variation and other host species in this shift. Temporal trends in environmental variables such as precipitation, soil moisture, temperature, and normalised difference vegetation index (NDVI) were analyzed in relation to anthrax occurrence (presence/ absence and counts). Additionally, correlations between host species’ densities and anthrax mortalities over time were examined. Anthrax cases in 1990 were concentrated in the central and northern regions of KNP(excluding Pafuri), primarily affected kudus; while subsequent mortalities affected mostly impala and were restricted to the far north, in Pafuri. Significant correlations were found between kudu anthrax mortality and a decrease in NDVI, average temperature, SPI-6 and SPI-12 (Standardised Precipitation Index in various time intervals. Conversely, anthrax occurrence in impalas was associated with a decline in SPI-3, and temperature rise, with increased mortality during the rainy season. Elephant density correlated negatively with kudu mortality, but a positive correlation with both impala mortality and impala density. The study concludes that environmental variables and species’ densities may alter the diversity and frequency of hosts exposed to *B*. *anthracis*. Climate extremes and alterations therein may exacerbate anthrax severity by modifying species susceptibility and their probability of exposure over time.

## Introduction

Environmental and climate variables [1–3] as well as the behaviour and densities of host species [4] have been identified as key transmission drivers for certain persistent pathogens in the environment. Host activities, which include foraging and movement behaviour, could significantly modify exposure risk to pathogens [5–8]. The changes in disease patterns over time are also linked to the behaviour of other host species in a shared ecosystem [9], the persistence of environmentally transmitted pathogens, and the populations of disease vectors [10]. Environmental factors, including precipitation [11–14], temperature [15, 16], and plant cover [17], are significant drivers of the occurrence of diseases, such as anthrax, and may therefore contribute to shifts in outbreak dynamics over time.

Anthrax is a multispecies animal disease caused by the *Bacillus anthracis* bacterium, chiefly recognised as a disease of mammals [18]. While primarily fatal to herbivorous wildlife and livestock, humans are susceptible to anthrax infections, and human cases occur largely due to the handling of infected carcasses, meat, and hides [19, 20]. *Bacillus anthracis* is an aerobic, non-motile, Gram-positive rod-shaped bacterium that produces endospores that persist in the environment [21] and are influenced by environmental factors. Rainfall, humidity, precipitation, temperature, and soil type all influence the survival of *B*. *anthracis* endospores in the environment and their availability to be taken up by a host [22]. In general, anthrax-endemic locations are found in warmer climates [16, 22]. Because anthrax is indirectly transmitted, the spread of *B*. *anthracis* relies on a range of factors, such as soil, water, vegetation, and host foraging behaviour [12, 23–29], insect mechanical vectors such as flies [23, 30–33], and scavengers.

Anthrax is endemic in the northernmost part of the Kruger National Park (KNP) in South Africa [25], where the most affected host species has been a browser, the greater kudu (*Tragelaphus strepsiceros*), contributing up to 75% of recorded cases from 1960s-1990s [12]. Outbreaks in KNP predominantly occur in decadal cycles, often associated with dry seasons or periods of droughts [12, 23, 25, 34, 35]. This disease trend has, however, shifted to predominantly affecting impala (*Aepyceros melampus*; up to 70% of cases from 1991–2015), a mixed grazing-browsing herbivore, with annual outbreaks, mainly in the wet season in the Pafuri region of KNP [5, 6, 33]. Grazers such as plains zebra (*Equus quagga*), buffalo (*Syncerus caffer*) and blue wildebeest (*Connochaetes taurinus*) have contributed only 10% of anthrax-related deaths in this park from 1990–2015 [6].

A linkage has been established between the dissemination of anthrax and high concentrations of blowflies (*Chrysomya spp*.) with outbreaks in southern Africa [36]. Blowflies feeding on carcasses transfer *B*. *anthracis* on the leaves of nearby trees and shrubs, potentially exposing susceptible animals to the bacteria as they browse on these plants [33, 36]. This environmental-blowfly transmission pathway has been postulated to be the primary means of transmission for wildlife species such as kudu and other browsing animals [28, 30, 33]. Impalas are observed to predominantly graze during wet seasons, while their browsing activity increases during dry seasons, typically foraging at heights below 1 meter. In contrast, kudus are known to forage up to 3 meters above ground level [37]. We thus hypothesise that changes in vegetation structure could indirectly alter browser exposure to *B*. *anthracis*.

Enhancing our understanding of ecosystem structure and trophic level interactions can highlight the impact of common species on ecosystem dynamics and resilience [9] and, in turn, extend to the interplay with disease dynamics within the ecosystem. Previous studies have reported a sharp drop in the number of big trees in KNP such as *Combretum apiculatum* (russet bushwillow), *Terminalia sericea* (silver cluster leaf), *Senegalia nigrescens* (knobthorn), *Sclerocarya birrea* (marula) and *Colophospermum mopane* (mopane), which constitute approximately 80% of the trees in the region [38]. While an earlier study suggested that elephants may not affect the abundance and diversity of tree species [39], a more recent study recorded notable declines in the park’s woody vegetation between 1960 and 1989 [40]. This study hypothesised that the sharp increase in elephant density might be a contributing factor to the tree population decline. The reduction in woody cover within KNP cannot be solely attributed to elephants; other species such as impala have been identified as drivers of change. Previous research indicates that impala plays a role in regulating the transition from shrubland to woodland, while elephants facilitate the transformation from woodland to shrubland [8]. This dynamic interaction is particularly significant for browsers such as kudu that depend on woodlands for foraging [41] and towards the anthrax-blowfly transmission pathway. Further investigation of the impact of other host species on anthrax transmission dynamics, particularly the shift from kudu to impala in KNP warrants considerable attention.

Variations in host density can influence disease occurrence, the decline in the population due to disease, and the spread of infection within individual hosts across a landscape affected by disease [42]. For a significant period, host density has been considered a crucial element in theoretical models due to its influence on contact rates [43]. Increased host density typically results in higher contact rates, which can enhance the transmission of infectious diseases [44]. Anthrax outbreaks in KNP have been attributed to species density [12], illustrating how variations in host population densities can directly impact the occurrence and spread of disease.

Density-dependent transmission, where the contact rates and thus transmission rates increase with host density, is a key concept in understanding the epidemiology of infectious diseases. This is particularly relevant in the context of anthrax outbreaks, as higher host densities can facilitate the spread of the bacterium through increased contact rates or environmental contamination [45]. Higher densities of susceptible species, such as impala, may lead to more frequent blowfly-mediated transmission events, thereby influencing the dynamics and persistence of anthrax outbreaks in these populations [25]. However, most studies focus on evaluating the influence of host density on disease transmission dynamics among interacting host species within the community. It is crucial to examine the influence of species’ density in the shift from kudu to impala in anthrax-related mortalities because density can play a significant role in the environmental-blowfly transmission pathway.

Climate change increases disease risk and pathogen exposure [46]. Effects include temperature changes, altered rainfall, storms, droughts, and floods [2, 47, 48], impacting vegetation, herbivores, carnivores, and scavengers. These events can contaminate water and food, and shift host and pathogen distributions [1–3], especially for *B*. *anthracis*. In South Africa, climate change threatens biodiversity, health, food security, and water resources [49]. Climate change alters temperature, rainfall patterns, and hydrological cycles, affecting biodiversity and agriculture [50]. Increased CO_2_ and nitrogen promote woody plant growth, affecting habitats for browsers [51], and altering species distributions and interactions [52]. Long-term environmental changes may affect host susceptibility and disease dynamics [46, 53]; therefore investigating the role of climate variables (rainfall and temperature) in host exposure and susceptibility to *B*. *anthracis* is thus crucial.

In this study, we investigated the possible drivers of the change in main anthrax host species from kudu to impala, including the roles of climate and environmental factors as well as the densities of other host species in KNP. Specifically, we evaluated if changes in environmental conditions (precipitation, temperature, soil moisture, NDVI, and SPIs) over time were correlated with changes in anthrax case numbers separately for each host species. Secondly, we investigated the role of host densities and host count by correlating animal density and count with anthrax mortality.

## Materials and methods

### Study area

**Fig 1:**
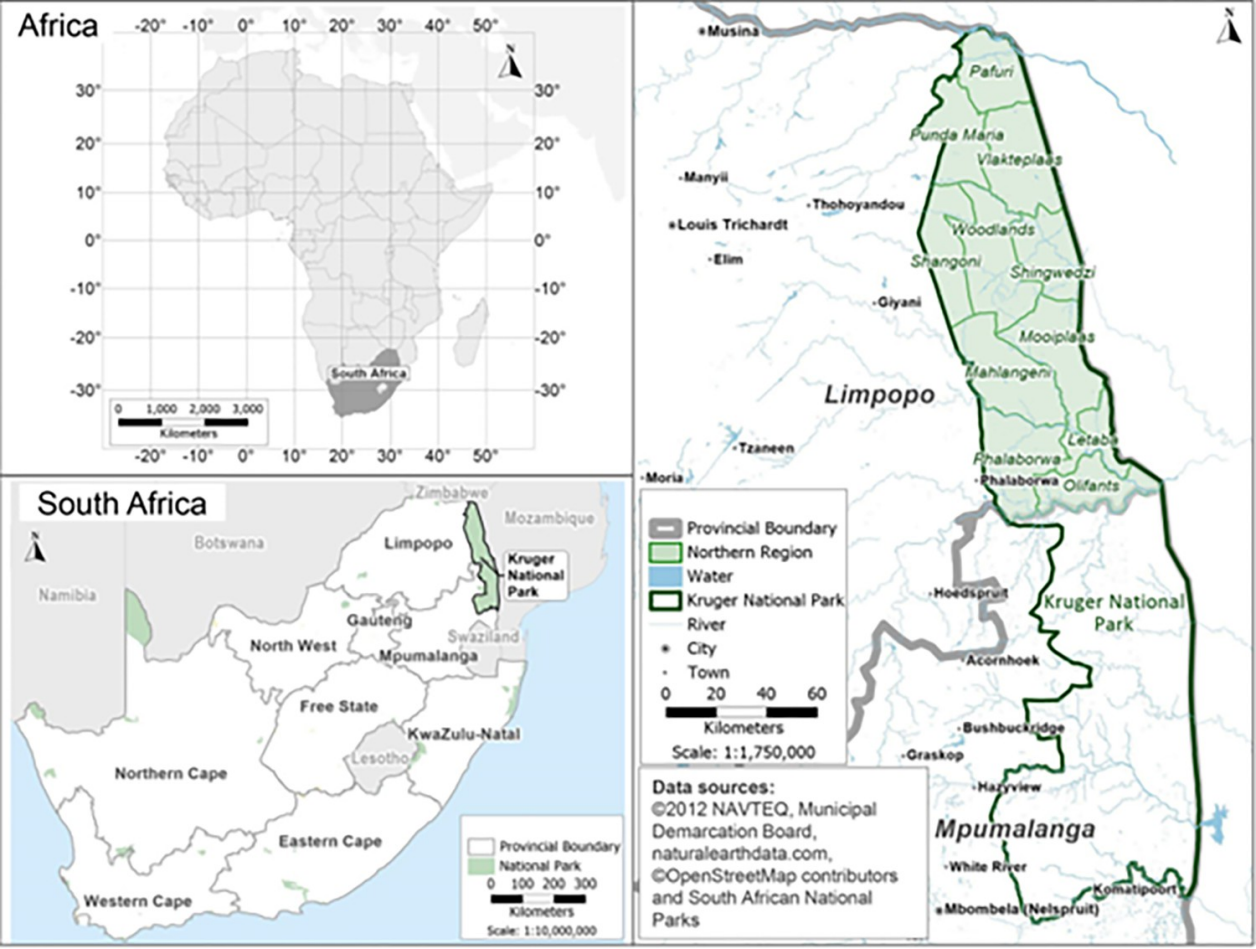
Map of study area in the northern region of Kruger National Park (KNP) in South Africa, showing central to northern areas in KNP where anthrax mortalities have been recorded (shaded in green). South Africa provincial and municipal map obtained from https://download.geofabrik.de/africa.html. KNP shape files were obtained from the South African National Parks under the project permission Ref: BMTA 006/22) and also from Navteq [71]. The Africa map was obtained from the natural earth data (https://www.naturalearthdata.com/downloads/).

This study was conducted in the Kruger National Park^,^ South Africa. The highest incidence of anthrax mortality in KNP is in the northern section (22.4206° S, 31.2296° E) extending from Pafuri in the north to the central region ending at Olifants River (Fig 1). This region of the park accounts for 88.4% of the anthrax mortalities from 1990–2015 [6, 15, 54]. This area is characterised by an average rainfall of 430 mm [55] and heavily wooded and grassland savannas [56]. The predominant tree species are *Combretum apiculatum* (russet bushwillow), *Terminalia sericea* (silver cluster leaf), *Senegalia nigrescens* (knobthorn), *Sclerocarya birrea* (marula) and *Colophospermum mopane* (mopane) [38]. The ecological seasons in KNP are classified as wet (December–March), early dry (April-July) and late dry season (August-November) [57, 58]. Kudu, impala and African buffalo (*Syncerus caffer*) are the predominant anthrax host species, and their habitat is largely woodlands with flood plains extending along the Limpopo, Luvuvhu, Shingwedzi and Olifants rivers. However, elephant mortalities have also increased in recent years [5].

### Ethics statement

This study was reviewed and approved by University of Pretoria Research Ethics Committee, Animal Ethics Committee (REC 049–21), Department of Agriculture, Forestry and Fisheries (DAFF) in South Africa (Ref 12/11/1/1/6 (2382SR)) in South Africa, South African National Parks (SANParks), South Africa (Ref: BMTA 006/22).

### Anthrax mortality data

Opportunistic passive mortality surveillance data (for kudu, impala and elephant) were obtained from the Skukuza State Veterinary services. Anthrax mortality was defined as samples confirmed positive through microscopy, culture, molecular detection of *B*. *anthracis* genetic markers as well as from symptoms pathognomonic of anthrax as prescribed by the World Health Organization, 2008. We used anthrax mortality data from 1990–1999 and 2010–2015 (omitting data from 2000–2009 because of poor surveillance following the prioritization of anti-poaching activities) after excluding data without georeferenced (Global Positioning System-GPS) positions to allow for standardization through time. Metadata recorded at the carcass sites include the date, georeference (GPS coordinates), locality, species, and sex. We used the number of cases in subsequent analyses and did not calculate prevalence due to the lack of consistent population estimates per species over time. The spatial extent of the analysis was the northern half of the park as cases occurred across the entire region even though most of the mortalities were concentrated around the Pafuri section.

### Environmental variables

We explored the associations between different environmental variables and the number of kudu and impala anthrax mortalities. Rainfall data were used to better understand relationships between precipitation and anthrax mortalities as well as to assess whether the change in rainfall patterns has affected kudu and impala differently. When considered annually, rainfall years were defined from July to June as described by [5]. The daily precipitation (rainfall) data were obtained from the Climate Hazards Group InfraRed Precipitation with Station data (CHIRPS), which is a quasi-global rainfall dataset that spans over 35 years, using a 5 x 5 square metre grid of the northern half (from the Olifants river) of KNP [59]. We also evaluated the relationship between anthrax mortalities and the Standardized Precipitation Index (SPI) which is an indicator for characterizing and indicating droughts and the probability of precipitation in any time scale and anthrax mortalities [60]. The SPI is a widely used metric globally to identify and describe meteorological droughts, which are extended periods of below-average rainfall in a specific area. SPI assesses precipitation anomalies at a particular location by comparing observed total precipitation over a designated accumulation period with the long-term historical rainfall data for that same period and location [61]. SPI values below ‒1.0 indicate rainfall deficits (drier than normal), whereas SPI values above 1.0 indicate an excess of rainfall. The magnitude of the drought increases as the SPI value decreases [61]. SPI-3 period is useful for evaluating drought on a short-term 3-month basis, while the 6- and 12-month accumulation periods are useful for evaluating drought on a longer-term basis [61]. As such, SPI longer than 3 months (6–12 months) can be utilized as a predictor of decreased stream flow and reservoir storage when estimated for medium accumulation periods.

As animal behaviour and foraging patterns may be affected by precipitation at different time lags, we hypothesised that the effect of precipitation on vegetation growth and hence kudu and impala behaviour may vary depending on the time scale investigated. We calculated separate SPI values that represent different time scales (3 months, SPI-3; 6 months, SPI-6; and 12 months, SPI-12). The SPI values were calculated from the precipitation CHIRPS data mentioned above, by using the SPEI function [62] in R [63]. We also investigated whether environmental variability (in terms of vegetative greenness) affected kudu and impala anthrax case numbers with vegetation greenness or condition estimated using Normalized Difference Vegetation Index (NDVI), which was obtained from the remotely sensed Landsat 5/7/8/9 at 30 m spatial resolution data through climate engine [64, 65]. As temperature has also been shown to be a significant driver of anthrax outbreaks [16, 22], we assessed the association between minimum temperature (Tmin), maximum temperature (Tmax), and average temperature (Tavg) with anthrax mortality. The temperature dataset was obtained from TerraClimate, which uses climatically aided interpolation, combining high-spatial resolution climatological normals from the WorldClim database [66].

### Host counts and density data

We used population estimates for kudu, impala, and elephant (1985–2015), from annual aerial surveys to evaluate patterns in host density in relation to anthrax mortality over time. To evaluate associations between anthrax mortality and host densities, animal count and density data were obtained from South African National Parks (SANParks) Scientific Services for the northern half of KNP. Aerial animal count data from 1985–2015 were recorded as total area counts (sampling method details in Kruger, Reilly [67]). Abundance estimates were performed using DISTANCE software in versions 4.0 and later as described by Thomas, Buckland [68], with data being binned into intervals in conjunction with conventional distance sampling.

Initial surveys from 1998 to 2000 found that distance sampling with 15% coverage of KNP was sufficient for producing population estimates of all larger ungulates that were adequately accurate (20% CVs) [69]. The east-west transect lines were deliberately spaced every 3’ of latitude (about 5.6 km apart) to make surveying more representative of the region [70]. The sampling intensity was raised to 22% from 2001 through 2003 by placing transects every 2’ of latitude (about 3.7 km apart). In 2004, the intensity was once more increased, this time to 27%, by flying higher and widening the transect. The 2004 data were not included in this paper since these modifications made it impossible to directly compare it to prior years. The coverage north of the Olifants River was raised to 28% (one transect per 1"36’ of latitude) in 2005 and 2006 using the original height and transect width and by adding 13 transects. The precision of sampling intensities was compared with ANOVA while the coefficients of variation (%) were determined in DISTANCE.

Elephant population abundance and densities in KNP were estimated using helicopter aerial survey data, which were conducted in August and September to correspond with the late dry season, when most animals are limited to areas near water. The survey followed the standardized technique that has been in use since 1977, with a pattern flown by helicopter along the rivers and important drainage systems, counting all elephants.

### Statistical analysis

#### Descriptive analysis of anthrax mortality for kudu, impala and elephants

Anthrax mortality count data for kudu, impala and elephant were plotted to understand the spatial and temporal distribution patterns of cases in central to northern KNP, aggregated by year between 1990–1999 (excluding 1992), the 2010–2015 periods, and according to ranger sections of KNP.

All maps were designed using Maptitude Geographical Interface system software (Version 2023 Build 5505 64-bit) with the South Africa country data package.

#### Trend analysis for NDVI, precipitation, SPIs, temperature, host densities and count

First, we performed time series analyses for the 1985–2015 period to understand whether there were significant temporal trends in NDVI, precipitation, SPI-3, SPI-6, SPI-12, average, minimum, maximum temperatures as well as the densities of elephant, impala and kudu. We analysed temporal trends in the monthly number of impala and kudu deaths over the 1990–1999 and 2010–2015 years. To address the issue of missing and non-linear data in population densities, we fitted a linear trend as an initial step. However, recognizing the complexity of the data, we employed the Augmented Dickey-Fuller test for a more robust analysis of these trends, incorporating a 12-month lag to reflect yearly seasonality (Dickey & Fuller, 1979). We used the tseries package in R for its comprehensive time series analysis tools, including the Augmented Dickey-Fuller test to assess stationarity (Trapletti & Hornik, 2020).

#### Generalised linear models for kudu and impala anthrax cases

Anthrax mortality data were zero-inflated, and thus we developed two models to analyse factors predicting anthrax-related deaths of kudu and impala from 1990–2015. We utilised Generalized Linear Models (GLMs) as the foundation for our analysis due to their flexibility in handling various types of response variables [72]. The binomial GLM was chosen to model the binary outcome of anthrax mortality (presence/absence), which is a standard approach for binary data. This model assumes that the logit of the probability of mortality is linearly related to the explanatory variables. For the count data, where the number of anthrax deaths exceeded zero, we applied a negative binomial GLM. This model is particularly suited for count data that exhibit overdispersion [73]. For each model, we tested nine explanatory variables that had a significant trend over time: NDVI, temperature maximal, precipitation, SPI3, SPI6, SPI12, season, and species’ densities. We also added the variable season (wet, early dry, late dry) and year as random variables as we know anthrax deaths are seasonal utilising the lme4 package for its capabilities in fitting mixed-effects models (Bates et al., 2015). Each variable was included as a random effect. We plotted the qqplot, residuals, and predictions to assess the model. We employed the MASS package for its implementation of the negative binomial GLM [74].

The StepAIC function in R, based on the Akaike Information Criterion (AIC), was employed for model selection using the MASS package. The AIC is a widely used criterion that balances model fit with complexity, penalizing the inclusion of unnecessary variables [75]. Model diagnostics were performed using quantile-quantile plots (qqplots) using the ggplot2 package [76], residuals analysis, and prediction accuracy checks to ensure the assumptions of GLMs were met and the models were well-fitted to the data.

#### Correlation between host density and anthrax mortality

As the best Generalized Linear Models performed poorly, probably due to omission of important variables, we examined relationships between the number of anthrax deaths, and the elephant, kudu and impala densities and count. While the animal census in KNP was conducted approximately every two years, survey methodology varied over time and the census years do not always correspond to the anthrax mortality surveillance years. To further explore the relationship between anthrax mortality and the densities of various host species, we performed Pearson correlation analyses using the base R statistical environment. This method is suitable for quantifying the linear relationship between two continuous variables [77].

Hence, due to the inconsistencies and absence of density data for some years, we considered 5 time points, and treated them like independent samples, so the correlation was following individual years 1990–1995; 1998; 1999; 2010, and the average over the 2011–2015 years. We aggregated the years 2011–2015 because of the absence of corresponding years with population count data from impala, kudu and elephant. We used the Pearson correlation between each pair of variables to determine their relationships at 5% significance level, using R version 4.1.2 [63].

## Results

### Distribution of anthrax mortality in northern KNP

**Fig 2:**
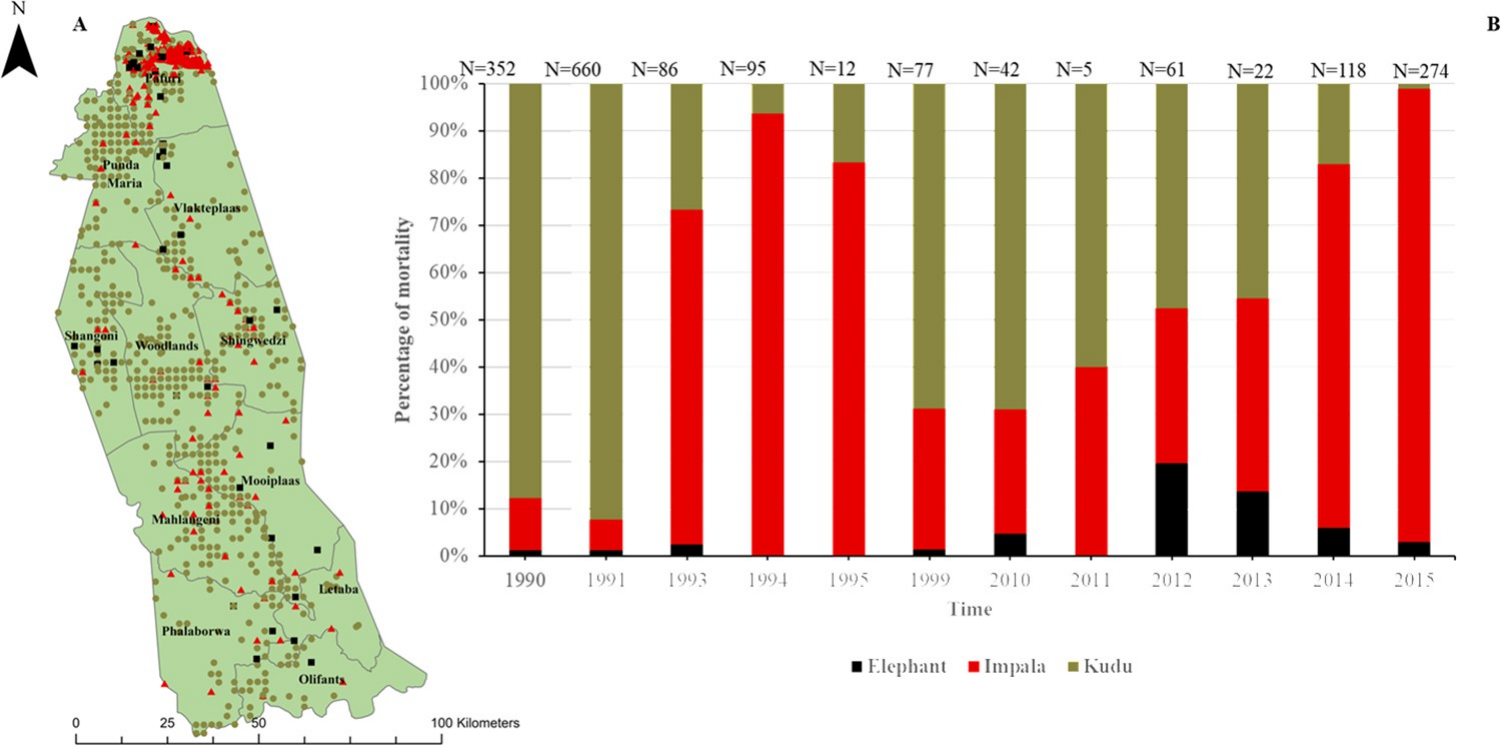
A) Anthrax mortalities in northern Kruger National Park (KNP), South Africa, in the three study species 1990–1995. A) Map showing the distribution of anthrax mortalities across the central to northern KNP. Coloured dots represent elephant (*Loxodonta africana;* black), impala (*Aepyceros melampus*; red), and kudu (*Tragelaphus strepsiceros*; tan) anthrax mortalities found across the different ranger sections (bold font) of the park. B) Stacked bar chart showing percentage of anthrax mortalities by species between 1990–2015 in the central to northern region of KNP: elephant in black, impala in red and kudu in tan The total number of mortalities per year is shown above each stacked bar graph. Municipal map obtained from https://download.geofabrik.de/africa.html. KNP shape files were obtained from the South African National Parks under the project permission Ref: BMTA 006/22) and also from Navteq [71].

**Fig 3:**
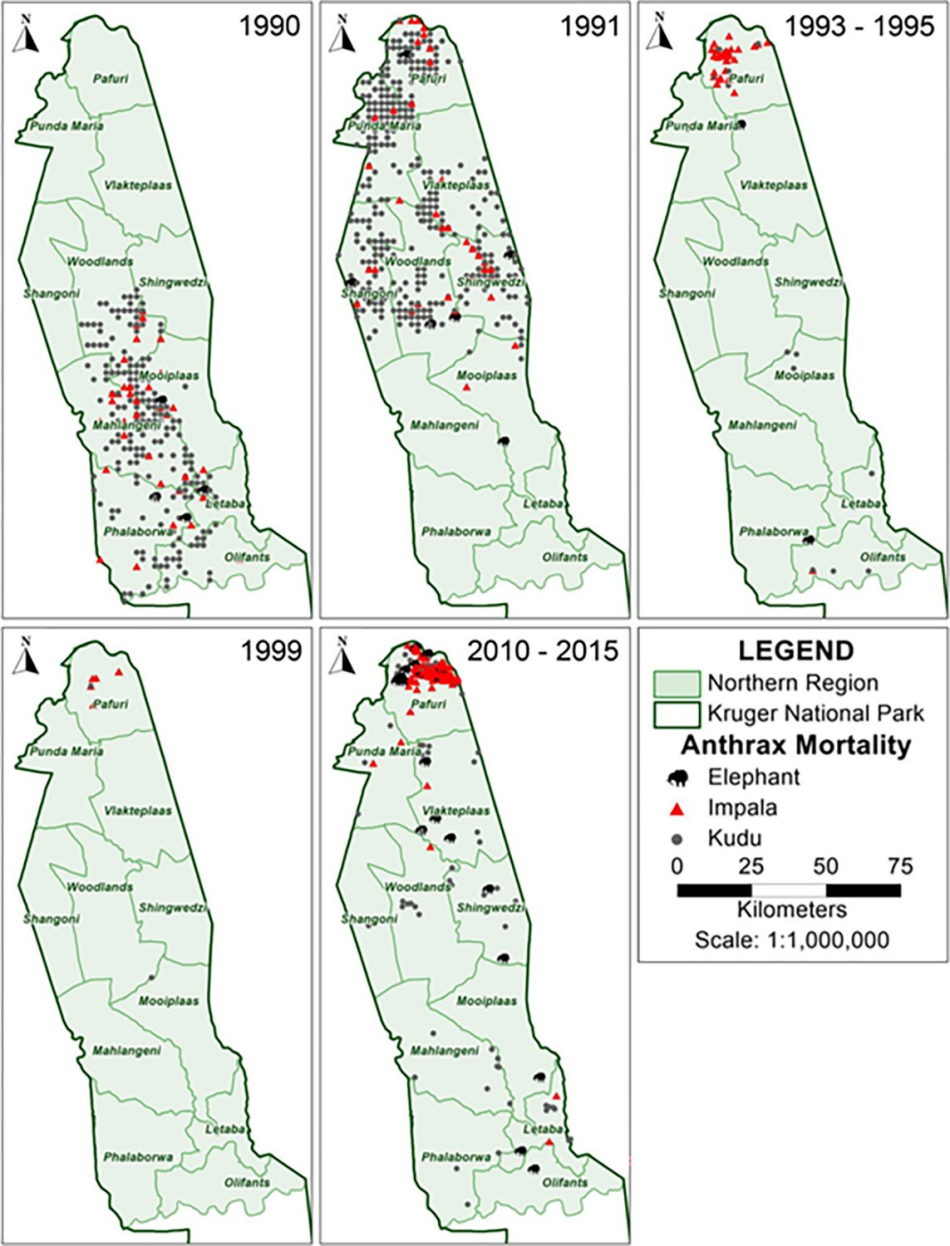
Map series showing the distribution of anthrax mortalities from 1990 to 2015. Animal species are indicated by icons, with the black animal silhouette representing elephant (*Loxodonta africana*), red triangle representing impala (*Aepyceros melampus*) and grey circle representing kudu (*Tragelaphus strepsiceros*) anthrax mortalities across ranger sections in the central to northern region of KNP. Municipal map obtained from https://download.geofabrik.de/africa.html. KNP shape files were obtained from the South African National Parks under the project permission Ref: BMTA 006/22) and also from Navteq [71].

Distributional patterns of anthrax mortality among kudu, impala and elephant in KNP demonstrated spatial and temporal differences. Most of the earlier anthrax mortalities in 1990–1991 were comprised of kudu (Fig 2) and were scattered across the entire central to northern region (from Olifants to Limpopo Rivers, Fig 3). Of the total anthrax mortalities for kudu, impala and elephants (*n* = 1809 from 1990–2015), kudu contributed 60.47% (*n* = 1,094), impala contributed 35.91% (*n* = 650) and elephant contributed 3.62% (*n* = 65). In 1990, mortalities occurred in the central part (between Olifants and Singwedzi Rivers) with no kudu anthrax mortality in the northernmost part (Pafuri, Punda Maria, Vlakteplaas sections; Fig 3) of the park. In 1991, kudu anthrax mortality in the Pafuri region was 19.38% of the total anthrax mortality observed that year in the three species (*n =* 118 out of 660 cases), and kudu anthrax mortalities that year were spread across the central to northern areas of the park from Mooiplaas northwards to Limpopo River (Fig 3). Of the total observed kudu-only anthrax mortalities across the central and northern parts of the park between 1990–2015, kudu in the Pafuri region constituted only 14.14% (*n* = 1094; Fig 2), showing that most of the kudu anthrax mortality over the years occurred outside the Pafuri region of the park (Fig 3).

The first impala anthrax mortality in Pafuri in this study was in 1991, with mortality in Pafuri constituting 25.58% of the impala anthrax mortality in that year, but this also represented only 1.67% (n = 11) of the total anthrax mortality for the three species in that year (Fig 2). But from 1991–1999 and 2010–2015, 93.63% (*n* = 612) of the total impala anthrax cases were found in Pafuri (Figs 2 and 3), whereas only 6.37% (*n* = 39) of impala cases occurred outside this region over the entire time period investigated (1990–1999 and 2010–2015). This shows that impala anthrax mortality in Pafuri following the reported case in 1991 has remained largely restricted to Pafuri (Fig 3).

For elephant, anthrax mortalities have been spread across the entire park with slight increases in case numbers from 2010–2015 in Pafuri, which accounts for 63.33% (*n* = 30) of the total elephant anthrax mortality in the central to northern parts of KNP (Fig 2).

### Trend analysis and association of host mortality with environmental, climate, and host variables

**Fig 4:**
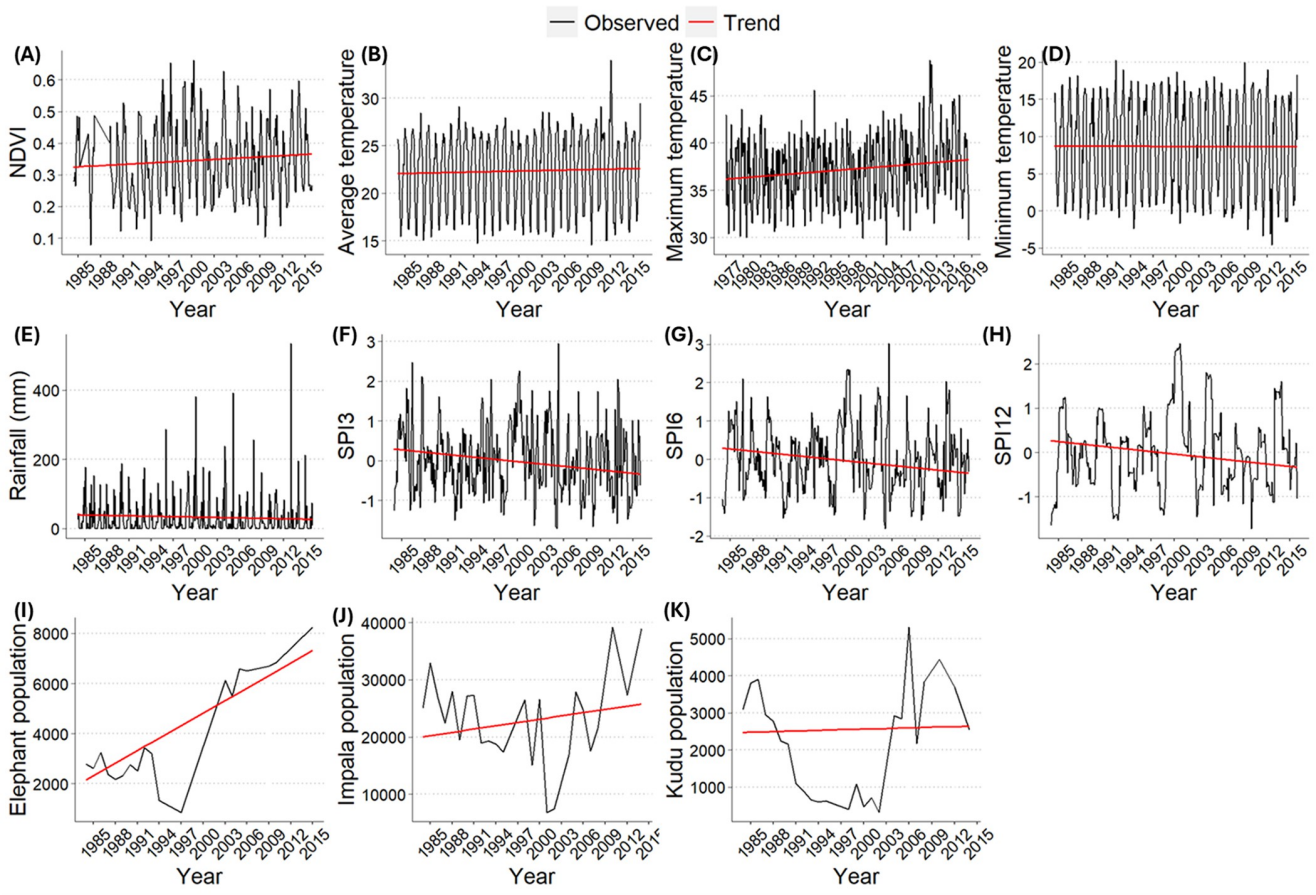
Charts showing time trend analyses of environmental, climate, and host variables (in red), against the observed data (in black) from 1980–2015 in northern Kruger National Park, South Africa. A) Normalized Difference Vegetation Index (NDVI), B) average temperature, C) maximum temperature, D) minimum temperature, E) rainfall in millimeters (mm), F) Standardized Precipitation Index (SPI) for 3 months (SPI-3), G) SPI for 6 months (SPI-6), H) SPI for 12 months (SPI-12), I) elephant (*Loxodonta africana*) density, J) impala (*Aepyceros melampus*) density, K) kudu (*Tragelaphus strepsiceros*) density. Significant trends are indicated with **.

The trend analysis showed a significant increase in NDVI and average and maximal temperature over time (Fig 4A–4C), however minimal temperature and precipitation did not show significant temporal trends (Fig 4D and 4E). There was a significant decrease in all the SPIs (Fig 4F–4H) showing a drying trend over the study period. Elephant density also significantly increased over time (Fig 4I). The time trend analyses showed that there was not a significant linear trend in impala (Fig 4J) or kudu (Fig 4K) densities over time.

Kudu anthrax mortalities were significantly more likely to occur during the dry season when the SPI-12 was high (*p* = 0.007) and NDVI low (*p* = 0.007; S1 Table). The number of kudu anthrax cases was significantly negatively associated with NDVI (*p* = 0.0047), lower average temperature (*p* = 0.0018), and elephant density (*p* = 0.0180), low-NDVI dry seasons are associated with more mortality than average dry seasons. On the other hand, kudu mortality counts were positively associated with SPI-6 and SPI-12 (*p*<0.0001; S2 Table), therefore, higher than average SPI in the previous wet season is associated with more kudu mortality. Presence/absence of impala mortality was linked to an increase in maximal temperature (*p* = 0.004), which is hotter than average condition, NDVI (*p* = 0.009), with a 12 month lag as in SPI-12 (*p* = 0.0004), and decreased kudu density (*p* = 0.0001; S3 Table), but the model performed very poorly (root mean square error = 4.48) and thus no significant conclusion can be obtained from it. However, impala mortality count was significantly associated with a decrease in SPI-3 meaning drier than average conditions in the 3 months preceding cases (*p*>0.0001), and an increase in elephant density (*p*<0.035), temperature (*p*<0.0157) and season (*p*<0.0009; S4 Table).

By examining the magnitude and significance of the beta estimates for kudu, the variables most strongly associated with anthrax mortality are SPI-12, NDVI, Seasondry, and Precipitation, with SPI-12 showing the highest significance. In the kudu mortality count model, SPI-12 remains the most significant, followed by the SPI-6, Precipitation, Tavg, NDVI, and elephant density. For impala, the most critical variables for anthrax mortality presence/absence are kudu density, SPI-12, NDVI, and Tmax, with kudu density being highly significant. In the impala mortality count model, the most significant predictors are the NDVI, SPI-12, elephant density, Tmax, and SPI_3, NDVI showing the highest significance.

### Correlation analysis of anthrax mortality and species density

Correlation analyses investigating associations between host species density and anthrax mortality for kudu, impala and elephants showed varying patterns (Table 1). There was a significant negative correlation between kudu anthrax mortality and elephant density (*p*<0.05; Table 1), as well as a negative, but non-significant, correlation with kudu density and anthrax mortality. Also, there was a significant positive correlation between kudu density and elephant density (*p*<0.01; Table 1). For impala, there was a significant positive correlation between impala anthrax mortality and elephant density, but a significant negative association between impala density and impala anthrax mortality. Further, there was a significant positive association between impala density and elephant density. For elephants, there was a significant positive correlation between their density and anthrax mortality (*p*<0.05; Table 1).

**Table 1:** Correlation table between anthrax mortality and animal density for kudu (*Tragelaphus strepsiceros*), impala (*Aepyceros melampus*) and elephant (*Loxodonta africana*).

	Kudu mortality	Impala mortality	Elephant mortality	Kudu density	Impala density	Elephant density
Kudu mortality		0.01	0.57	-0.36	0.29	-0.51*
Impala mortality			0.35	0.40	-0.41*	0.5*
Elephant mortality				0.59	-0.03	0.59*
Kudu density					-0.59*	0.81*
Impala density						0.40*
Elephant density						

The table presents the Pearson correlation coefficients with pairwise deletion. Statistical significance is denoted with an asterisks

(*) at *p*<0.05. A value of -1 represents a perfect negative correlation while a value of 1 represent perfect positive correlation.

## Discussion

Seasonal or interannual changes in resource availability influence animal habitat selection, leading to fundamental ecological shifts that can alter pathogen exposure risks among wildlife populations over time [78]. In this study, we explored the influence of environmental variables and host density on anthrax mortality patterns within a community of herbivorous mammals. Our aim was to elucidate the potential mechanisms behind a shift in the primary anthrax host species from kudu to impala in KNP. Over the study period, vegetation greenness (NDVI) and temperature both increased, while the association between drought measures (SPIs 3,6 and 12) and anthrax mortality between kudu and impala varied. Furthermore, kudu and impala anthrax mortalities were significantly associated with changes in environmental variables and with elephant density. We hypothesised that changing climate trends, coupled with the indirect effects of elephant density on other herbivores, may contribute to changes in anthrax outbreak dynamics in the KNP ecosystem. These changes could have resulted in either a temporary or permanent shift in anthrax occurrence to primarily impala cases and wet season outbreaks.

A range of factors play a role in the introduction and dissemination of pathogens in new populations, which in turn can have significant impacts on disease transmission dynamics, hosts’ exposure and susceptibility [79]. Anthrax in KNP was historically associated with dry seasons or droughts, with explosive outbreaks occurring on a roughly decadal cycle [23, 25, 34, 35]. During the initial phase of the study spanning from 1990–1991, kudus were the predominant species in anthrax outbreaks in the central to northern part of KNP. This period coincided with a major drought event in KNP [80]. The 1990 anthrax outbreak was widespread across the central and northern parts of the park and largely impacted kudu. However, in 1991, the outbreak was predominantly in the northern region, specifically the Pafuri section of the park. This outbreak primarily affected kudu with few impalas affected. After 1991, subsequent outbreaks predominantly occurred in the Pafuri section with impala being the primary species affected. We hypothesise that this shift in outbreak dynamics may signify a shift to a new host population, primarily composed of impala. Furthermore, this change could have also been spatially constrained to the Pafuri region, where impala density is higher and kudu density lower compared to other parts of the park. This spatial distribution suggests a potential association between host populations density and the localization of anthrax outbreaks [81].

Outbreaks in regions of KNP appear to be influenced by rainfall and the hydroclimatic conditions of the location [12]. Changes in climate and environmental variables have been shown to drive or exacerbate host susceptibility to diseases and exposure, thereby affecting disease dynamics [53]. Previous findings showed that seasonal variation significantly affects anthrax occurrence in different regions [11, 13, 28, 82]. In line with such findings, our study confirmed that kudu mortality was higher in the dry season, especially for the earlier outbreaks (1990–1991). We found that kudu mortality was associated with a decrease in NDVI. This seasonal pattern is consistent with the hypothesis that ingesting drier leaves increases the chances of getting wounds around the oral cavity when foraging, facilitating pathogen invasion [27]. In contrast, impalas (mixed grazing-browsing herbivore) have been found previously to be affected during annual outbreaks that occur primarily in the wet season [33]. According to Nsoh, Kenu [13], most of the epidemics in grazers happen at the closing of the dry season and the onset of the rainy season, which could force animals to feed closer to the ground and as such, potentially increase the likelihood of these animals acquiring anthrax. However, grazing closer to the ground can happen in the wet season too, with higher soil contact in the wet season in grazers of Etosha National Park, Namibia [83]. Overall, changes in diet by season seem to affect exposure risk.

Impalas are mixed feeders; in the rainy season they are predominantly grazers with graze constituting 91.8% and browse 8.2% of their diet, while in the dry season, their diet is comprised of 66.4% graze and 33.6% browse [37, 84, 85]. This suggests that impala feeding patterns may be sensitive to short- and medium-term moisture conditions, as confirmed by the observed association between impala anthrax mortality and SPI3 in our study. Previous studies have similarly established correlation between soil moisture and NDVI [86, 87]. This means that impala would be grazing more during the wet-season timing of cases. Our time series analysis showing a decline in SPI-3 together with the significant negative association between impala mortality and SPI-3, suggests climatic changes may have contributed to increasing anthrax mortalities in impala over time. Most impala anthrax mortalities occur during early wet seasons which may coincide with regeneration of vegetation at carcass sites and this mortality continues for a long period of time with eventual decay of exposure dose [29, 45, 88].

Host population density has been reported to be one of the major drivers of an anthrax outbreak in KNP [23]. However, our study revealed a negative correlation between kudu mortality and their density, suggesting that kudu mortality may not be primarily influenced by their own density but by other factors, such as the density of other species within the ecosystem. Although one might expect anthrax mortality to decrease kudu density, the frequency of population counts was insufficient to investigate population changes at the scale of an outbreak. Also, kudu anthrax mortality was correlated with an increase in elephant density. Understanding the interaction between host species and their ecosystem is a great determinant of understanding how their behaviour influences the survival and resilience of that system, which in turn influences the dynamics of an outbreak.

Although the environmental blowfly transmission pathway was not explicitly tested in this study, it likely contributes significantly to the observed patterns of anthrax mortality among kudu and impala in KNP. Blowflies are key vectors of anthrax, and their activity is influenced by environmental factors that also affect host species behaviour and habitat use [25, 32, 89]. With the negative relationship between kudu anthrax mortalities and, potentially due to the impact of elephants on woody cover, we hypothesise this in turn affects blowfly habitats [32]. Reduced tree cover might decrease blowfly populations or alter their distribution, thus impacting anthrax transmission to kudu, which rely on these woody areas for browsing. Also, elephants’ impact on browse in the dry season can reduce the available browse for kudus or shift blowfly roosts lower, making them accessible to impala. This increases impala’s exposure risk. These dynamics underscore the complex interplay between host density, environmental changes, and vector activity in disease transmission, suggesting that further research on the role of blowflies could provide valuable insights for managing anthrax outbreaks in KNP.

Impala anthrax mortality, on the other hand, was correlated with an increase in elephant density. Further, there has been a significant increase in impala population in KNP over time. Impala has been shown to have a significant influence on woody vegetation. Mature trees are felled by elephants by pushing them over or debarking them [90] while impala predate tree seedlings of up to 1.5 metres in height [91]. Destruction of woodlands by elephants is often believed to be self-limiting as, following the relocation of the elephants from that particular site, the trees tend to regenerate [8]. However, Stokke and du Toit [92] hypothesised that elephants in Chobe National Park, Botswana, who browse primarily in the 1–3 m zone above ground, cannot account for the nearly entire disappearance of regenerated knobthorn and other once numerous trees along the Chobe riverbank at present, suggesting a role played by other herbivores such as impala in limiting recovery of woody species [93].

Increasing impala densities have been linked with declining densities of preferred woody recruits [94]. It has been hypothesised that impala may play a key role in shaping the landscape by establishing and maintaining a dynamic mosaic of patches. This hypothesis was supported by Moe, Rutina [93] who reported that elephants facilitated the transformation from woodlands to shrublands, while impala browsing prevented the transition from shrubland to woodlands. Elephants create browsing “lawns” for mesoherbivores like impala and kudu [95], although a higher density of impala in the northernmost region of the park may result in more mortalities compared to the kudu population. Furthermore, another study reported a relationship between impala anthrax mortality and bush encroachment, where an impala population crash-released seedlings from browsing pressure, allowing same-aged woody stands to establish [96]. This demonstrates how diseases like anthrax can influence herbivore-vegetation dynamics, with ripple effects across the herbivore community, as observed in the study conducted in East Africa [96].

Finally, this study showed that kudu anthrax mortalities were higher when elephant density was lower. For impala, there was a positive correlation between anthrax mortality and elephant density, between impala density and elephant density, and also between impala anthrax mortality and density. We, therefore, hypothesise that the anthrax shift from kudu to impala, and from dry to wet seasons, could be a temporary pendulum that could swing back to kudu, as hinted at in what seems like cycles of kudu density and mortality (seen in Figs 2 and 4K). Secondly should the impact of elephant and impala on woody cover be abated, the disease burden may return to kudu. The adaptability of herbivores to changing conditions plays a crucial role in determining the permanence of these behavioural changes and their disease implications.

## Conclusion

In conclusion, the findings of this study shed valuable light on the shifting dynamics of anthrax outbreaks in KNP. It becomes evident that comprehending the distinct roles played by various host species and the environment is crucial for unraveling the complexities of anthrax outbreaks. The temporal analysis of mortalities revealed a significant shift in the primary host species affected by anthrax, transitioning from predominantly kudu to impala over time. Temporary or permanent shifts in environmental and climate variables, as well as host-associated variables such as foraging behaviour and density, could increase or decrease the chances of exposure to *B*. *anthracis* which in turn may cause a temporary or permanent shift in hosts’ exposure risk. Remarkably, the density of individual host species did not emerge as a significant factor influencing anthrax-related mortalities. Instead, the density of other species seemed to exert an indirect influence, which may be driven by impacts on vegetation. Such an indirect influence may contribute to increased impala exposure to *B*. *anthracis* and a decreased risk for kudu. In essence, this study underscores the intricate interplay between host species, environmental factors, and changing anthrax dynamics in the study area.

## Limitation of the study

1. The lack of tree cover data across KNP constrained a comprehensive understanding of the impact of elephants and impalas on vegetation.
2. Inconsistencies in the sampling method for density data across all species also compromised the efficacy of the employed models.
3. The relatively sporadic population counts were insufficient to capture changes in population dynamics, given their scale mismatch with the mortality data.
4. The decrease in surveillance efforts due to the prioritization of anti-poaching activities hindered a thorough investigation of all patterns, resulting in missing data for some years.

## Suggestions for future research

Future studies to examine the role of herbivorous host species, vegetation dynamics and animal density in wildlife disease transmission may be of immense benefit to understanding anthrax transmission dynamics in host communities. Secondly, exploring the potential effect of the shift from woodlands to shrublands on the *B*. *anthracis* blowfly-browse transmission pathway can improve understanding and prediction of shifts in anthrax outbreak dynamics. We also suggest that future research consider the spatial implication of the environmental variables on anthrax mortality using the geographically weighted models. Lastly, we suggest that future studies investigate the roles of other species, particularly grazers such as zebra and buffalo, in driving the observed patterns in kudu mortalities. These species could impact resource competition and habitat use, thus affecting kudu density and mortality rates.

## Supporting information

S1 Data (ZIP)

S1 Table Binomial generalized linear model table for the presence/absence of anthrax mortality for only kudu (*Tragelaphus strepsiceros*) with the presence/absence as the response variable and season, Normalized difference vegetation index (NDVI), standardised. (DOCX)

S2 Table Negative binomial model table for the anthrax mortality count for only kudu (*Tragelaphus strepsiceros*) with the count data as the response variable and season, Normalized difference vegetation index (NDVI), standardised precipitation index (SPI), and precipitation as predictor variables. (DOCX)

S3 Table Binomial generalized linear model table for the presence/absence of anthrax mortality for only impala (*Aepyceros melampus*) with the presence/absence as the response variable and season, Normalized difference vegetation index (NDVI), standardised precipitation index (SPI) 3 and 12, year of mortality and kudu (*Tragelaphus strepsiceros*) density (TS_density) as predictor variables. (DOCX)

S4 Table Negative binomial model table for the anthrax mortality count for only impala (*Aepyceros melampus*) with the count data as the response variable and season, Normalized difference vegetation index (NDVI), standardised precipitation index (SPI), maximal temperature, elephant (*Loxodonta africana*) density (LA_density), year and kudu (*Tragelaphus strepsiceros*) density (TS_density) as predictor variables. (DOCX)
