# Supplementary material for: Roles of host and environment in shift of primary anthrax host species in Kruger National Park
Source: PLoS One. 2024 Dec 6;19(12):e0314103. doi: 10.1371/journal.pone.0314103 (PMC11623471; doi:10.1371/journal.pone.0314103)
Supplement: S1 Table — (DOCX) [file pone.0314103.s002.docx]

Table S 1: Binomial generalized linear model table for the presence/absence of anthrax mortality for only kudu (*Tragelaphus strepsiceros*) with the presence/absence as the response variable and season, Normalized difference vegetation index (NDVI), standardised

Coefficients:

|  | Estimate | Standard Error | z-value | Pr(>\|z\|) |
| --- | --- | --- | --- | --- |
| Intercept | -3.044e+03 | 1.463e+02 | -2.080 | 0.0375 |
| Seasondry | 1.473e+02 | 9.917e-01 | 1.486 | 0.0100* |
| NDVI | -1.097e+01 | 4.090e+00 | -2.683 | 0.0073** |
| SPI-12 | 1.6090e+00 | 5.3983e-01 | 2.690 | 0.0072** |
| Precipitation | 1.681e-02 | 9.553e-03 | -1.760 | 0.0784 |

---

Signif. codes: 0 ‘***’ 0.001 ‘**’ 0.01 ‘*’ 0.05 ‘.’ 0.1 ‘ ’ 1

(Dispersion parameter for binomial family taken to be 1)

Null deviance: 97.074 on 71 degrees of freedom

Residual deviance: 76.933 on 63 degrees of freedom

AIC: 90.933

Number of Fisher Scoring iterations: 5
