# Supplementary material for: Roles of host and environment in shift of primary anthrax host species in Kruger National Park
Source: PLoS One. 2024 Dec 6;19(12):e0314103. doi: 10.1371/journal.pone.0314103 (PMC11623471; doi:10.1371/journal.pone.0314103)
Supplement: S2 Table — (DOCX) [file pone.0314103.s003.docx]

Table S 2: Negative binomial model table for the anthrax mortality count for only kudu (*Tragelaphus strepsiceros*) with the count data as the response variable and season, Normalized difference vegetation index (NDVI), standardised precipitation index (SPI), and precipitation as predictor variables.

Coefficients:

|  | Estimate | Standard Error | z-value | Pr(>\|z\|) |
| --- | --- | --- | --- | --- |
| Intercept | 5.95959 | 1.05307 | 5.659 | 1.52e-08*** |
| NDVI | -8.44163 | 2.98379 | -2.829 | 0.00467** |
| SPI_6 | 0.4670 | 0.1377 | 3.392 | 0.000014 *** |
| SPI_12 | 2.46440 | 0.48211 | 5.112 | 3.19e-07*** |
| Tavg | -0.4874 | 0.1560 | -3.125 | 0.001777 ** |
| LA_density | 0.4291 | 0.1814 | 2.365 | 0.018046 * |
| Precipitation | -0.02522 | 0.00802 | -3.145 | 0.00166** |

---

Signif. codes: 0 ‘***’ 0.001 ‘**’ 0.01 ‘*’ 0.05 ‘.’ 0.1 ‘ ’ 1

(Dispersion parameter for Negative Binomial(0.2406) family taken to be 1)

Null deviance: 111.529 on 71 degrees of freedom

Residual deviance: 55.221 on 68 degrees of freedom

AIC: 290.78

Number of Fisher Scoring iterations: 1

Theta: 0.2406

Std. Err.: 0.0590

2 x log-likelihood: -280.7760
