# Supplementary material for: Roles of host and environment in shift of primary anthrax host species in Kruger National Park
Source: PLoS One. 2024 Dec 6;19(12):e0314103. doi: 10.1371/journal.pone.0314103 (PMC11623471; doi:10.1371/journal.pone.0314103)
Supplement: S3 Table — (DOCX) [file pone.0314103.s004.docx]

Table S 3: Binomial generalized linear model table for the presence/absence of anthrax mortality for only impala (*Aepyceros melampus*) with the presence/absence as the response variable and season, Normalized difference vegetation index (NDVI), standardised precipitation index (SPI) 3 and 12, year of mortality and kudu (*Tragelaphus strepsiceros*) density (TS_density) as predictor variables.

Coefficients:

|  | Estimate | Standard Error | z-value | Pr(>\|z\|) |
| --- | --- | --- | --- | --- |
| Intercept | -8.354e+02 | 2.176e+02 | -3.839 | 0.0001*** |
| Tmax | 1.6874 | 0.2560 | 2.125 | 0.00376 ** |
| NDVI | -1.020e+01 | 3.926e-00 | -2.599 | 0.0093** |
| SPI_3 | -7.822e+01 | 4.960e+01 | -1.577 | 0.1148 |
| SPI_12 | 2.268e+00 | 6.401e-01 | 3.543 | 0.0004*** |
| Year | 4.230e-01 | 1.100e-01 | 3.847 | 0.0001*** |
| TS_density | -1.991e-03 | 5.402e-04 | -3.684 | 0.0002*** |

---

Signif. codes: 0 ‘***’ 0.001 ‘**’ 0.01 ‘*’ 0.05 ‘.’ 0.1 ‘ ’ 1

(Dispersion parameter for binomial family taken to be 1)

Null deviance: 97.074 on 71 degrees of freedom

Residual deviance: 63.871 on 66 degrees of freedom

AIC: 75.871

Number of Fisher Scoring iterations: 5

Root mean square error = 4.48
