# Supplementary material for: Roles of host and environment in shift of primary anthrax host species in Kruger National Park
Source: PLoS One. 2024 Dec 6;19(12):e0314103. doi: 10.1371/journal.pone.0314103 (PMC11623471; doi:10.1371/journal.pone.0314103)
Supplement: S4 Table — (DOCX) [file pone.0314103.s005.docx]

Table S 4: Negative binomial model table for the anthrax mortality count for only impala (*Aepyceros melampus*) with the count data as the response variable and season, Normalized difference vegetation index (NDVI), standardised precipitation index (SPI), maximal temperature, elephant (*Loxodonta africana*) density (LA_density), year and kudu (*Tragelaphus strepsiceros*) density (TS_density) as predictor variables.

Coefficients:

|  | Estimate | Standard Error | z-value | Pr(>\|z\|) |
| --- | --- | --- | --- | --- |
| Intercept | -6.097807 | 1.345847 | -4.531 | 5.88e-06 *** |
| NDVI | 2.986190 | 0.838951 | 3.559 | 0.000372 *** |
| SPI_3 | 0.308030 | 0.151794 | 2.029 | 0.042432 * |
| SPI_12 | 1.390e+00 | 3.681e-01 | 3.775 | 0.00016** |
| Tmax | 1.840e-01 | 7.054e-02 | 2.609 | 0.00908** |
| LA_density | 7.149e-04 | 2.457e-04 | 2.910 | 0.00362** |
| Year | 2.493e-01 | 5.910e-02 | 4.219 | 2.46e-05*** |
| TS_density | -4.189e-04 | 2.935e-04 | -1.427 | 0.15333 |

---

Signif. codes: 0 ‘***’ 0.001 ‘**’ 0.01 ‘*’ 0.05 ‘.’ 0.1 ‘ ’ 1

(Dispersion parameter for Negative Binomial (0.6831) family taken to be 1)

Null deviance: 106.840 on 71 degrees of freedom

Residual deviance: 70.506 on 65 degrees of freedom

AIC: 311.03

Number of Fisher Scoring iterations: 1

Theta: 0.683

Std. Err.: 0.166

2 x log-likelihood: -548.423
